# Losing Your Gut Feelings. Intuition in Depression

**DOI:** 10.3389/fpsyg.2016.01291

**Published:** 2016-08-23

**Authors:** Carina Remmers, Johannes Michalak

**Affiliations:** ^1^Vivantes Wenckebach Clinic – Clinic for Psychiatry, Psychotherapy and PsychosomaticsBerlin, Germany; ^2^Department of Clinical Psychology, University of HildesheimHildesheim, Germany; ^3^Department of Clinical Psychology, Witten/Herdecke UniversityWitten, Germany

**Keywords:** intuition, depression, automatic processes, decision-making, mood

## Abstract

Whereas in basic research, intuition has become a topic of great interest, clinical research and depression research in specific have not applied to the topic of intuition, yet. This is astonishing because a well-known phenomenon during depression is that patients have difficulties to judge and decide. In contrast to healthy individuals who take most daily life decisions intuitively (Kahneman, 2011), depressed individuals seem to have difficulties to come to fast and adaptive decisions. The current article pursues three goals. First, our aim is to establish the hypothesis that intuition is impaired in depression against the background of influential theoretical accounts as well as empirical evidence from basic and clinical research. The second aim of the current paper is to provide explanations for recent findings on the depression-intuition interplay and to present directions for future research that may help to broaden our understanding of decision difficulties in depression. Third, we seek to propose ideas on how therapeutic interventions can support depressed individuals in taking better decisions. Even though our knowledge regarding this topic is still limited, we will tentatively launch the idea that an important first step may be to enhance patients’ *access* to intuitions. Overall, this paper seeks to introduce the topic of intuition to clinical research on depression and to hereby set the stage for upcoming theory and practice.

## Introduction

In many situations, individuals judge and decide without long reflections about the problem at hand. Despite the lack of long deliberation, the decisional and judgmental outcomes are often smart and satisfactory (Gigerenzer, 2007). In other words, we sometimes know what is right even if, we cannot explain why. In many situations, this is because, we use our intuition. Even though intuition is a cognitive capacity that influences many decisions and subsequent actions in daily life (Kahneman, 2011), it has received little attention so far within clinical research on cognitive processes and decision-making in depression. This seems unfortunate because individuals with depression often report to have difficulties to come to decisions (American Psychiatric Association [APA], 2013). Converging with its preceding versions, the fifth edition of the Diagnostic and Statistical Manual of Mental Disorders (DSM-5) includes indecisiveness as a diagnostic criterion for Major Depressive Disorder (American Psychiatric Association [APA], 2013). A question that may follow from this often reported phenomenon is whether intuitive decision-making is impaired during depression. Research on this hypothesis, however, is scarce.

## Aims of the Current Paper

The current paper addresses three objectives. Our first aim is to develop the hypothesis that intuition is impaired in depression and that considering intuition in the scope of depression research may have important theoretical and practical consequences. The second aim of the article is to point to methodological issues and open questions. Against the background of novel findings on the depression-intuition interplay, we will propose directions for future research and specific ideas as to how research on intuition may broaden our knowledge regarding decision difficulties in depression. The third aim of the current paper is to adopt a practical and therapeutical point of view by addressing the question how decision-making of depressed patients may be enhanced. Given that the overall knowledge regarding the interplay between depression and intuition is still limited, we will raise novel questions rather than giving concluding answers on this topic and hereby seek to set the stage for future research.

## Decision-Making During Depression

When facing a decision, patients suffering from an acute episode of Major Depression often cannot make up their mind what to do. The depressive mind is narrowed to a tunnel vision in which patients tend to circle around the same (negative) pieces of information. This processing style is called rumination (Nolen-Hoeksema et al., 2008; Lyubomirsky et al., 2015). Rumination means to repetitively over-think the causes and consequences of one’s situation or mood state (Nolen-Hoeksema et al., 2008). It has been shown that a ruminative self-focus predicts and prolongs depression and that it impairs problem solving (see Lyubomirsky et al., 2015 for a review). Thus, rumination may interfere with intuitive processes as it fosters a narrow and analytical information processing style (Watkins and Teasdale, 2004).

Research has shown that depressed individuals take poor decisions (Leykin and DeRubeis, 2010) or no decision at all (Okwumabua et al., 2003) – a phenomenon they severely suffer from American Psychiatric Association [APA] (2013). Moreover, it has been shown that depressive symptomatology is significantly associated with reduced search for information (Leykin et al., 2011). Individuals with higher levels of depression further report that ambiguities and uncertainties remain unresolved after they take a decision (Leykin et al., 2011). Furthermore, higher levels of depression are associated with reduced perception of existing resources (e.g., assistance of other people; own talents) and reduced satisfaction with decisions (Leykin et al., 2011). Individuals with depression have a high risk to feel uncertain about decisions they have taken (Stacey et al., 2008). Moreover, depressed individuals report more anticipatory regret (Schwartz et al., 2002; Monroe et al., 2005). Whereas anticipatory regret may serve as a warning mechanism that protects a person from bad decisions (McCormack et al., 2015) it seems that depressed individuals experience such high levels of anticipatory regret that this results in passivity and inaction. In line with this, depressed patients report to have less confidence and self-esteem regarding their decision-making capacities (Leykin and DeRubeis, 2010) and tend to take decisions that have had negative outcomes previously (Leykin et al., 2011). Thus, patients with depression seem to have difficulties to learn from prior decision-making experiences and tend to use maladaptive strategies repeatedly.

The above-mentioned shows that decision-making during depression seems to be aﬄicted with difficulties. The question, which component of decision-making is impaired during depression remains open. It should be noted that there are a number of factors that may be related to decision-making difficulties such as impaired reasoning capacities in depression (Radenhausen and Anker, 1988; Sedek and von Hecker, 2004; Perham and Rosser, 2012; Jung et al., 2014), lacking appreciation of information (Hindmarch et al., 2013) or limitations in working memory capacities (Channon and Baker, 1994) and increased ruminative processes (Nolen-Hoeksema et al., 2008). However, in the following, we aim to address the topic of potentially impaired intuitive processes because intuition is an important and – amongst healthy samples – often used decision-making tool (Gigerenzer, 2007; Kahneman, 2011) with highly adaptive features, especially in personally relevant, complex decision making situations (Kuhl, 2001; Topolinski and Strack, 2009a).

## What is Intuition?

Since researchers investigate decision-making and judgment, there has been a fascination for the topic of intuition. Ancient philosophers used the term *nous* (greek: *noein)* to refer to the ability of human beings to grasp what is real or true. Nous (or *noesis*) is often translated with ‘good sense’ or ‘intuition’ and it stands in contrast to rational, conscious reasoning. In this ancient definition, intuition is understood as a vehicle by which one can get aware of what one already knows. As such, it may allow people to get access to pre-existing knowledge. Intuition further received considerable attention in the scope of psychoanalysis. Jung (1921) conceptualized intuition as a means by which a person can see *the bigger picture*. According to Jung (1921), intuition strives for new possibilities in what is objectively given. Intuition is the vehicle that automatically operates as soon as no other psychological function is able to find a way out of a complex situation (Jung, 1921). Along this line, for Jung, intuition is about discovering – a facet that still applies to current conceptions of intuition (Bowers et al., 1990).

Modern social and cognitive psychology operationalize intuition as a specific product in which puzzle pieces are quickly put together. Intuitions result from information processes that operate fast, associative and unconsciously (Kahneman, 2011). Prior experiences and their mental representations, build the basis for intuitive judgments and decisions. Thus, operating like a pattern completion mechanism, it appears that intuitive judgments are related to prior learning experiences and arise through unconscious holistic spreading processes (Sadler-Smith, 2008). They are often experienced as if they had come out of nowhere and enable individuals to detect coherences and patterns (Kahneman and Klein, 2009). Intuitions are typically described with the phenomenon of *knowing something without knowing how* (Epstein, 2010).

In order to directly elicit and measure intuition in the laboratory, researchers developed paradigms such as the semantic coherence task, a well-established experimental paradigm developed by Bowers et al. (1990). In the semantic coherence task intuition is operationalized as the sudden perception or realization of coherence based on unconscious activation spread within associative networks (Bolte et al., 2003; Bolte and Goschke, 2005). During the task, participants see triads of words. Each word triad consists of three words, presented in a stacked format on a computer screen. Participants are asked to judge intuitively whether the presented word triad shares a common denominator (e.g., SALT DEEP FOAM; all words are associated to the solution concept SEA; coherent triad) or whether the triad consists of randomly selected words (DREAM BALL BOOK; no common denominator; incoherent triad). The intuitive performance is reflected by the degree to which participants can differentiate between coherent and incoherent word triads without being able to explicitly name the solution word (which would be indicative for insight and not intuition; Bolte and Goschke, 2005; Topolinski and Strack, 2009a,b,c; Topolinski and Reber, 2010). It has been shown that healthy participants are generally able to detect semantic coherence above chance level (Bolte and Goschke, 2005). They know when a triad is coherent, without being able to explicitly name the underlying solution word. This is even shown in experimental designs, in which participants have less than 3 s for their decision, a time window during which the operation of explicit processes is very unlikely (Bolte and Goschke, 2005). Thus, the semantic coherence task operationalizes and measures intuition by assessing the activation of information (solution word) which is not consciously accessible (Bolte et al., 2003; Bolte and Goschke, 2005).

According to the continuous model (Bowers et al., 1990), intuitions arise from a gradual two-stage process. Within the first stage, information spreads and accumulates. This results in the activation of an associated network. Because of its activation, the mnemonic network is processed more fluently (Topolinski and Strack, 2009a,c), which in turn is accompanied by subtle positive affective changes (Topolinski and Strack, 2009a,c for empirical demonstrations of processing fluency). It is during this first stage, *the guiding stage*, when a person may experience the feeling of coherence – an intuition (Bowers et al., 1990). If the unconscious activation spread of coherent information exceeds a certain threshold the initial intuitive feeling of coherence may evolve into the explicit representation of the solution. This second stage, in which a person can explicitly reason about the decision or action taken within the guiding stage, is called the *integrative stage* (see Zander et al., 2015 showing distinct brain activation patterns for each stage of the intuition generation process).

The theoretical conception of a continuous two-stage progress from intuition to explicit insight, allows us to hypothesize at which stage impairments may occur in individuals who have little intuitive capacities. For example, the inability to take decisions based on intuitive processing may be attributable to impairments at very early stages of the intuition generation process, such as reduced spreading activation within the semantic networks. However, it is also conceivable that intuitive impairments may occur because individuals are not able to make use of subtle positive affective cues, normally elicited by coherence perception (Topolinski and Strack, 2009a,b). On a later stage, it may be that an individual has the intuition but does not use it because of low confidence in his or her decisional abilities. Overall, it becomes clear that the conceptualization of intuition generation as a two-stage process may have important consequences regarding further theorizing.

## The Advantages of Intuition

For a long time, intuition was the black box of modern experimental psychology (Catty and Halberstadt, 2008) and initial research programs in this field focused on instances in which non-deliberate, heuristic problem solving strategies lead to erroneous and suboptimal outcomes (Tversky and Kahneman, 1974). However, within the past decades, research on potential advantages of intuitive decision-making has received particular attention. Studies in the scope of the Naturalistic Decision Making Paradigm (Klein, 1998, 2008), for example, demonstrated that subjects from various professional backgrounds such as firefighters, doctors, chess players, nurses, and judges use their intuition in complex situations and under high stress and time pressure. Especially in situations in which rational-analytical processing is not possible (e.g., under stress or uncertainty) and in case of high experience with the problem at hand, intuitions can lead to impressively adaptive outcomes. When large amounts of information need to be encoded, intuitive decisions bear better outcomes and lead to more diagnostic judgments than extensive reasoning. A vivid demonstration of this has been shown by Betsch et al. (2001). In their study, participants were given large amounts of information concerning the numerical increases and decreases of five hypothetical shares. Seventy-five units of information were briefly presented on a computer screen. Even though participants could not explicitly tell what, for example, the average money returns were, they had developed a gut feeling of what the best and worst options were.

Subsequent studies bolstered the idea that relying on intuitive hunches is especially useful when the problem at hand is complex in nature (Dijksterhuis, 2004; Dijksterhuis and van Olden, 2006; see also Wilson and Schooler, 1991; Topolinski and Strack, 2008) and that deliberate processes such as searching for solutions or memorizing may even impair decision-making performance (Topolinski and Strack, 2008). Also in the context of social cognition, intuition has received considerable attention (Lieberman, 2000). Studies that operationalized intuition with the semantic coherence task found that intuitive processing seems to be especially relevant for the enactment of affiliation motives (Maldei et al., under review) and that intuitive performance is positively associated with meaning in life (Hicks et al., 2010).

Moreover, it has been shown that people are also more satisfied with decisions that were based on their gut feeling. In their seminal study, Wilson et al. (1993) let participants choose a poster that they could take home. Subjects could choose either intuitively or after thinking through the reasons why they liked or disliked each alternative. Results revealed that subjects in the rational-reasoning condition were less satisfied with their choice when asked about 3 weeks after the experimental session compared to subjects who chose a poster intuitively. Reduced levels of satisfaction in the analytical group may have occurred because analytic processing typically abstracts from the emotional and personal meaning of a decision at hand (Kuhl et al., 2015). In other words, analytic processes reduce the complexity of a problem by breaking ambiguous information down to one aspect that is important in a particular situation (Dijksterhuis, 2004; Kuhl et al., 2015). This is of advantage for logical problem solving but of disadvantage when the problem includes divergent aspects that need to be considered (e.g., solving a complex personal problem; interpersonal relationships; dealing with an illness; see Kuhl et al., 2015). For the latter problem type, intuitive decision-making seems to be advantageous.

Also in the context of personality psychology intuitions that are based on holistic and associative processing sequences are conceived as highly adaptive. More specifically, Personality Systems Interaction Theory (PSI; Kuhl, 2000, 2001), distinguishes between *low-level* and *high-level* intuitions. Low-level intuitions help people to execute concrete actions and typically arise under high levels of positive affect (Kuhl, 2001). They are guided by a system called *intuitive behavior control.* One of the ontogenetically earliest observation of such processes is the automatic imitation and contagion of emotional expressions in newborn children (Meltzoff and Moore, 1994). So whereas low-level intuitions help to implement intentions and to enact automatized behavioral programs, *high-level* intuitions derive from what PSI theory calls *extension memory*, a system that stores all experiences of a person and that integrates new information (Kuhl, 2001; Kuhl et al., 2015; see Lieberman et al., 2004 for neuropsychological evidence for intuition-based self-knowledge). The extension memory operates on the basis of unconsciously operating processes of activation spread, which enable a person to effortlessly include a vast amount of information regarding experiences, needs and goals simultaneously into the decision-making process (Kuhl et al., 2015). Thus, high-level intuitions are conceptualized as feelings or hunches in which diverging aspects of the self can be integrated. Intuitions help people to reconcile many – maybe even conflicting – aspects of a decision at hand and lead hereby to adaptive and helpful outcomes even when a person has not explicitly *thought* about all relevant aspects.

Altogether, the foregoing illustrates that the ability to make use of high-level intuitive processes may lead to adaptive outcomes in complex situations and connects us to ourselves in an integrated manner. In the following, we will thus further elaborate our main assumption of impaired intuition in depression by referring to influential theoretical accounts and empirical demonstrations from basic psychology.

## Theoretical and Empirical Indications for Impaired Intuition in Depression

Even though – normally – intuitions guide us through every-day life, there seem to be psychological states in which individuals are less intuitive and therefore less able to come adaptive decisions without long reflections. On the one hand, research has focused on external factors intuitive processes may depend on, such as time pressure or complexity of the problem (Klein, 1998, 2008). On the other hand, there are intra-individual conditions under which it is more or less likely that people will use their intuition. The question is thus, within which psychological states people easily decide intuitively and when they are blocked and unable to decide out of the belly. Because Major Depression is an affective disorder that is most and foremost characterized sustained negative mood, we will refer to empirical evidence from basic research on the interplay between mood and cognition in order to consolidate our assumptions in the following.

### Intuition and Mood

Regarding the question how people’s intuitive capacities are associated with their current mood state, considerations originating from affect-as-information-theory (Schwarz, 2002) and broaden-and-build theory (Fredrickson, 2001) provide important insights. According to these accounts, associative, flexible information processes needed for intuitions to develop, are more likely to operate under positive mood. Indeed, it has been shown that positive mood makes individuals find unusual (but reasonable) associations and fosters categorizations of material in a more flexible manner (Isen, 2001). The effects of positive mood on problem solving, flexibility and innovation are observable in a broad field of settings and among various populations (Isen, 2001). Most importantly for the current thrust, it has robustly been found that positive mood fosters the activation of remote semantic associations (Isen et al., 1985, 1987; Estrada et al., 1994; Fredrickson and Branigan, 2005) and that participants’ intuitive coherence judgments benefit from positive mood (Bolte et al., 2003; Balas et al., 2012). In addition, being in a positive mood makes it more probable to make use of feelings and intuitive hunches in the decision-making process (see affect-as information theory; Schwarz and Clore, 2007). Converging with this, there are several studies showing that individuals are more likely to rely on their intuitions when they are in a positive mood (Bless et al., 1990; Elsbach and Barr, 1999; Ruder and Bless, 2003; King et al., 2007) and intuitions themselves are accompanied by subtle positive affective cues (Topolinski and Strack, 2009a). Thus, positive mood enlarges our thought-action repertoire, widens the associative field and makes us consider more and new information (Csikszentmihalyi, 1990). As a result, individuals approach and explore their environment during positive mood states and consequently engage in activities (Diener and Diener, 1996; Fredrickson, 2001).

Negative mood states, in contrast, signal that the environment is problematic. This in turn narrows the thought-action repertoire (Fredrickson, 2001). Consequently, more analytical and systematic decision-making approaches are selected and flexible processing needed for intuitions to develop are inhibited. In line with this, affect-as-information theory (Schwarz, 2002), posits that negative mood states such as sadness foster cognitive analytic reasoning which makes individuals attend to few details rather than the bigger picture. Thus, whereas positive affectivity cues top-down processes, negative affectivity prompts bottom-up, data-driven and item specific processing (Clore et al., 2001; Clore and Storbeck, 2006). Converging with this, an influential study showed that in happy moods, participants match geometric figures on the basis of global similarities whereas in sad moods, subjects tend to match figures on the basis of local similarities (Gasper and Clore, 2002). Consequently, it is assumed that intuitive processes are impaired during negative mood states, because negative mood fosters analytic reasoning. Baumann and Kuhl (2002) investigated the interplay between intuition, affect and affect regulation ability and found that intuitions of semantic coherence were impaired by negative affect in participants who reported to have difficulties to down-regulate negative mood states. In contrast, intuitive performance was not impaired by negative mood in participants who were generally successful in down-regulating negative affective states (Baumann and Kuhl, 2002).

From a clinical perspective those findings are worth noting, as one of the main features of psychological disorders and especially Major Depression is the sustained experience of negative affectivity as well as the inability to down-regulate dysphoric mood states. Thus, enduring states of negative affectivity as well as the inability to experience positive affective states may be aspects of depression that inhibit open and flexible ways of processing information needed for intuition. To summarize, the assumption of impaired intuitive processing during depression is substantiated from several different theoretical perspectives.

### Depression and Intuition: Preliminary Findings

In the following, we will present three recent studies that have empirically tested the hypothesis of impaired intuition in depression. We will outline the study designs as well as findings of these three studies. Moreover, we will critically discuss the pattern of results and will then conclude which future studies should be done in order to further elucidate the interplay between depression and intuitive decision-making. The first study that has investigated intuition in depression (Remmers et al., 2015a) compared the intuitive performance of depressed inpatients (*n* = 29) to a healthy control sample (*n* = 27). Both samples were comparable in terms of gender distribution, while the depressed sample being slightly younger than the control group. To assess intuition, the well-established intuition measure described above, namely the semantic coherence task, was used. Results revealed that depressed inpatients were less able to detect semantic coherence than healthy control participants. In addition, depressed patients who fulfilled criterion A8 of the DSM-5 (American Psychiatric Association [APA], 2013), reflecting patients’ difficulties to think, concentrate and decide, had significantly lower intuitive accuracy than patients without those symptoms. Thus, this first study on intuitive performance during depression supported the hypothesis of impaired intuition in depressed patients.

Two follow-up studies aimed to replicate the finding that semantic coherence intuitions are impaired in depression and to generalize this finding to another intuition measure. In their first study, Remmers et al. (2016a) used a sample of depressed patients (*N* = 39) from a day-clinic. To replicate the finding of impaired semantic coherence detection, patients’ severity of depressive symptoms measured with the Beck Depression Inventory (BDI-II, Beck et al., 1996) was correlated with their performance in the semantic coherence task. To generalize the impairment in intuitive processing to another intuition measure, patients further completed the *visual coherence task* (Bowers et al., 1990; Bolte and Goschke, 2008; Topolinski and Strack, 2009c) which is similar to the semantic coherence task because it operationalizes intuition as fast, non-analytical coherence detection. However, the tasks differ in terms of stimulus type as in the visual coherence task participants see blurred pictures (instead of word triads presented in the semantic coherence task). One half of the stimulus pool is coherent because it contains distorted meaningful but very rarely explicitly identified pictures. For the other half of the stimuli the pixel information of the coherent pictures is rotated to such a degree that no meaningful gestalt is preserved. Thus, coherent as well as incoherent pictures contain the same pixel information but they differ in their arrangement. During the task, subjects are asked to judge whether the presented picture is coherent (depicting a real object) or incoherent (depicting no object). Similar to the semantic coherence task, it has been shown that participants are able to differentiate between coherent and incoherent pictures without being able to explicitly name the depicted pictures (Bowers et al., 1990). In their study, Remmers et al. (2016a) found in line with the study of Remmers et al. (2015a) that higher levels of depression were associated with less intuitive accuracy in the semantic coherence task. However, findings regarding the visual coherence task were against the initial hypothesis. Patients with higher levels of depression showed *enhanced* ability to detect visual coherence. Notably, there was a near zero correlation between the two intuition measures across the sample.

In order to explore the unexpected finding that visual coherence detection is enhanced in patients with higher levels of depression, the authors conducted a second study in which they compared the performance in the visual coherence task of depressed inpatients (*n* = 27) to a matched healthy control sample (*n* = 30). Similar to the study design of Remmers et al. (2015a), the diagnostic status of subjects was determined with the SCID interview. Results revealed that depressed patients did not only perform as good as healthy subjects, but that they outperformed the healthy control sample in discriminating coherent from incoherent blurred pictures. Granted that both measures assess the same construct namely intuition (see discussion on this below), it may tentatively be concluded that that for depressed individuals, processes underlying visual and semantic coherence detection are distinct from each other and that only language-based semantic intuitions seem to be impaired in depression. Visual coherence detection in contrast seems to profit from depressed mood. However, given the preliminary nature of these results future research should replicate these findings before drawing firm conclusions.

### How to Explain Detrimental and Beneficial Aspects of Intuition during Depression?

The novel dissociation between semantic and visual coherence intuitions during depression raises questions regarding the differential decisional consequences of depression and regarding the construct validity of intuition measures. Even though it has previously been postulated that successful performance in the semantic as well as in the visual coherence task results from equivalent processes, this assumption needs further investigation. For example, the near zero correlation between the semantic intuition index and the visual intuition index in Remmers et al. (2016a) raises doubts to whether both tasks measure the same construct. Furthermore, the deleterious effect of negative mood on coherence intuitions has only been shown for semantic coherence intuitions so far (Baumann and Kuhl, 2002).

Specific stimulus features and the processes needed for successful performance may explain the dissociation between depressed patients’ performance in the semantic and visual coherence tasks. A core difference between the two tasks used in Remmers et al. (2016a) is that one is based on visual processing whereas the other requires language-based, semantic processing. It has been assumed that – despite this difference in stimulus type – the two tasks measure the same construct, namely intuitive coherence detection (e.g., Topolinski and Strack, 2009a). However, the current pattern of findings regarding this capacity during depression suggests that the differences outweigh the commonalities between the tasks – at least as far as individuals with depression are concerned.

First, the finding that language-based intuitions are impaired, whereas visual intuitions are not, may be related to empirical evidence showing that biased responses in implicit memory tasks are only consistently found in depression for tasks that require processing of the *meaning* of stimuli (Watkins, 2002). Implicit memory tasks that require the attention to *perceptual* features, in contrast, are not biased during depression. Referring these findings to the results in Remmers et al. (2016a) it may thus be that particularly semantic coherence intuitions are impaired, as they require semantic meaning processing, whereas blurred pictures in the visual coherence task, do not and are therefore intact.

Along this, line, studies using magnetoencephalography (MEG) to investigate neural mechanisms underlying intuitive coherence perception are worth noting in elaborating the idea that semantic and visual coherence intuitions may be distinguished regarding underlying mechanisms and processes needed for successful performance. Horr et al. (2015) found that the orbitofrontal cortex (OFC) serves as a crucial integrator of incomplete stimulus input for semantic as well visual intuitions. However, there seems to be a striking difference in terms of temporal dynamics. Whereas in visual coherence detection, the OFC is one of the earliest regions that showed differential activation (Horr et al., 2014), OFC activation was comparably delayed in semantic intuition. In line with the foregoing, the authors point to conceptual difference between the two tasks. Visual coherence intuitions are specific to one sensory domain and based on low-level stimulus features which can directly be integrated by the OFC to a coarse holistic representation of the pixel information. In contrast, for semantic coherence intuitions, higher-level semantic processing needs to take place prior or parallel to the spreading activation process that signals coherence or incoherence, because each word of the word triad itself is a meaningful concept that needs encoding, respectively (Horr et al., 2015).

Furthermore, the dissociation between semantic and visual intuitions in depression may be related to the phenomenon that patients with depression tend to get caught in circles of rumination (see Watkins and Teasdale, 2004). Rumination operates largely language-based and it may be suspected that during depression the language-based processing mode is under high loads which may become evident in poor performance in tasks that require this capacity.

Another important task-specific particularity that should be discussed is that the detection of a Gestalt in the visual coherence task requires the isolation of an object within a stimulus. As such, successful performance in the visual coherence task requires that subjects attend to what is already there (the object within the blurred picture). From the angle of PSI theory this process may be assigned to what Kuhl (2000) calls the *object recognition system*. Importantly, this system is specialized in isolating elements from the context. It benefits from negative mood and fosters analytic-detailed processing on the one hand, but impairs holistic processing and self-compatibility checking on the other hand (Kazén et al., 2014). In line with this, it has been shown that subjects with emotion regulation difficulties are better to detect spelling errors in words (detail-oriented attention; isolating elements from the context) when they are in a negative mood compared to subjects who do not have difficulties in emotion-regulation (Kazén et al., 2014). In the semantic coherence task subjects focus on what is there, too: the three words written on the screen. However, in contrast to the object within the blurred picture in the visual coherence task, which is present during the task, the solution word (the common denominator) is not present (on the screen) in the semantic coherence task. Successful performance in the semantic coherence task thus requires letting the attention move away in order to integrate and finally use activated associations in the following judgment. Unlike the detection of an object within the blurred picture, this processing sequence may be assigned the *extension memory* (Kuhl, 2000), a system that fosters the integration of single elements (DEEP SALT FOAM) into a coherent whole (SEA) via high-level intuitive holistic processing sequences and it is connected to the integrated self. Thus, in line with the theoretical assumptions in the foregoing, this extended memory system including the parallel-holistic, flexible processing sequences that it relies on seems to be impaired during depression.

Finally, yet importantly, the findings of enhanced visual coherence judgments during depression may further be embedded into research showing that negative mood – in general – fosters detail-oriented and early visual processing (Bocanegra and Zeelenberg, 2009). For example, Phelps et al. (2006) found that participants’ contrast sensitivity is enhanced after viewing fearful faces. Furthermore, negative affective states have been shown to foster *spatial* working memory capacities whereas they impair *verbal* working memory capacities (Gray, 2001; Storbeck, 2012).

Concluding, a fine-grained analysis of stimulus features as well as of cognitive and emotional processes required for successful task performance can help to understand in how far different tasks eventually measure the same or distinct outcomes and how different task characteristics interact with psychological processes. From the current evidence, it may be concluded that depressed individuals have impairments in intuitions that rely on flexible, associative processes of *semantic* spread, but that depression might have no or even a beneficial effect on visual processes and visual gestalt perception. If these findings were consolidated in future studies, important practical implications may be concluded. For example, in therapeutic interventions it may be considered that depressed individuals have difficulties to recur on holistic semantic associations when solving problems. Supporting therapy sessions with visually based material, may thus be helpful in supporting patients to *see the bigger picture* and integrate information in a holistic manner.

However, for the moment, we think that conclusions should be drawn with care as the empirical basis is not sufficiently robust. Even though current findings suggest that in some instances intuitions are enhanced in depression whereas in others they are impaired, we think that a definitive conclusion would be premature. For example, we cannot conclude from the current studies whether impairments in other faculties such as analytical processes have influenced the operation of intuitive processes in the current studies. Upcoming research would do well in examining the interplay between intuitive processes and rational-analytic processes that may also be impaired and biased in depression (Beevers, 2005). In addition, future research should first of all elucidate the construct validity of the intuition tasks. Moreover, it should be examined to what extent the operationalization of intuition used in the former studies is related to depressed individuals’ decision-making styles in daily life. On the basis of these considerations, we will outline suggestions of future research that seeks to further elucidate the interplay between intuition and depression in the following.

## Directions for Future Research

### Which Mechanisms Underlie Intuitive Decision-Making in Depression?

The investigation of intuition and depression is still in an early phase. Concluding from current findings it seems important that future research first of all elucidates whether different intuition tasks effectively measure the same psychological phenomena. Furthermore, from the perspective of a continuous conceptualization of intuition (Bowers et al., 1990), future research should explore at what stage within the intuition generation process impairments occur. First, it should be explored whether the underlying process of semantic spreading activation is impaired in depression or whether this is intact, which would become obvious in successful performance in semantic priming tasks (see Topolinski and Strack, 2009a,c). In a next step it should be examined whether the impairment in intuitive performance is attributable to patients’ low confidence in their intuitive hunches (see for example Rolison et al., in press for a study on the effects of anxiety and reduced confidence on decision-making). If underlying processes of activation spread were shown to be intact in depression, and intuitive performance deficits mostly stem from low confidence levels, this would have important implications for therapeutic interventions that may consequently be directed to enhance patients’ trust in their intuitive capacities. Moreover, it should be explored whether activation spread is negatively biased in depression. This could be examined by using affectively laden word triads. One assumption may be that negative word triads are processed more fluently in depression, which would result in better intuitive accuracy for negative stimuli compared to positive stimuli (see Topolinski and Strack, 2009a for stimulus pool).

It should further be explored whether intuition deficits in depression are related to the diminished ability of depressed individuals to experience positive affect (Heller et al., 2009; Joormann and Vanderlind, 2014). This would be important to study as intuitive hunches have shown to be accompanied by subtle positive affective changes (Topolinski and Strack, 2009a,b) and intuitive decision-making itself is boosted by emotional information (Bolte et al., 2003; Lufityanto et al., 2016). Along this line, a recent study has found that especially people with affect regulation difficulties benefit from positive mood when taking intuitive decisions (Maldei and Baumann, 2015). However, depressed individuals may have problems to make use or even experience these positive affective cues needed for intuitive decisions. In other words, whereas in healthy people intuitive decisions just *feel right*, depressed patients may lack the ability to experience such positive feelings of coherence. This in turn may lead to less favorable decisions or no decision at all. Investigating these ideas would provide important insights on why depressed individuals struggle to come to decisions that feel right.

Moreover, future investigations would do well in assessing also effortful, analytical decision-making capacities of depressed patients. It would be of interest to examine how impairments in one capacity influences the other. For example, it should be explored whether intuitive processes are related to limitations in reasoning or working memory capacities. In addition, it should be explored to what extent the generation of irrelevant thoughts or ruminative processes impair intuitive decision-making, as for deliberate reasoning it has been shown that irrelevant thoughts elicited by negative mood impair performance (Perham and Rosser, 2012). In addition to these ideas, it would be interesting to investigate in future studies which neurophysiological impact antidepressants exhibit on unconscious processes of coherence detection. Altogether, there is a set of research questions resulting from the current empirical evidence on intuition in depression that are specific to the experimental tasks used in former studies (Remmers et al., 2015a; Remmers et al., 2016a).

Apart from these specific issues that concern well-established intuition measures such as the visual and semantic coherence tasks, upcoming research should further continue to explore intuitive capacities in depression by using measures that tap into other facets of intuition (Sinclair, 2011). For example, investigating intuitions based on stimuli that are more self-relevant may be especially important in order to increase ecological validity of empirical findings (Lieberman et al., 2004). This line of research would take into account that intuition is highly influenced by experience as it is ‘nothing more and nothing less than recognition’ (Kahneman and Klein, 2009, p. 520). Thus, even though intuitions are inter-individually comparable in terms of the processes they are based on (i.e., associative, unconscious, fast) people can differ regarding the content of these processes and the products that results from them. It thus becomes evident that some intuitions, such as semantic coherence intuitions assessed with the semantic coherence task are inter-individually comparable (most participants would agree that SEA SALT FOAM are all semantically connected to SEA) whereas others are largely idiosyncratic as persons may differ in their associative network and memory contents that are activated in certain situations (Lieberman, 2000; Lieberman et al., 2004). In line with this, evidence from neurophysiological research found distinct brain activation patterns for self-representations that are based on intuition (Lieberman et al., 2004). Biases in this domain would provide important insight, especially because intuition-based self-presentations are likely to change slowly and are relatively insensitive explicit feedback from others (Lieberman et al., 2004). Moreover, using more self-relevant stimuli for intuitive decision-making is important because we do not know to what extent intuition assessed with experimental paradigms such as the semantic coherence task relate to daily life intuitive decision-making.

Along this line, it would be of interest to differentiate between low-level and high-level intuitive processing suggested by PSI theory (Kuhl, 2000). Future research would do well in elucidating how low-level intuitive processing sequences that are related to automatized behavioral programs and that help to put plans into action are affected by depression. Moreover, and importantly, it would be of interest to examine how the activation or inhibition of self-regulatory systems such as the extension memory and intuitive behavior control system interact with each other within depressed patients and to what degree they play a role in predicting the onset of depressive episodes.

### Investigating Real-Life Decision-Making

Investigations that track idiosyncratic decision-making profiles of depressives or vulnerable subjects would help to understand *how* subjects decide when facing major or minor daily life decisions such as whether to accept a job offer or whether to go out meeting friends. Do they go with their intuition? Or do they reflect analytically about these issues? Do depressed individuals have decision difficulties in complex situations in which intuitions may help? Or does indecisiveness also occur for rather simple decisions, in which no high loads of information has to be integrated? To answer these questions, experience sampling methods may constitute a usable option as they can assess decision-making modes more directly by prompting subjects to render reports many times a day (Larson and Csikszentmihalyi, 1983; Hektner et al., 2006). Future studies could hereby also investigate in which decision-making *areas* (work, relationship, leisure time, health) patients report more or less difficulties. In a nutshell, to obtain a more precise picture of *how* individuals suffering from depression decide in daily life would inform our understanding of decision-making difficulties and may broaden our understanding regarding intuition in depression.

Another method that may be used for this thrust are retrospective reports assessed via interviews or survey questionnaires (Klein, 1998; Dane and Pratt, 2009). These methods allow participants to describe how they approached a decision-making problem and researchers to assess factors such as the complexity of the problem and the mood state prior and after the decision taken. Retrospective reports may further inform us when individuals with depression tend to take functional or dysfunctional decisions and whether the decision was grounded on intuitive or rational processes or both. It should be noted, however, that despite the advantage of high ecological validity retrospective reports are limited in terms of accuracy. For researchers it would be difficult to control whether decisions were actually made intuitively (see Dane and Pratt, 2009 for a discussion on this).

Last but not least, research should examine the etiological role of high-level intuitive capacities. For example, from a clinical perspective it would be of interest to explore whether the impairment in intuitive capacities in specific and decision-difficulties more generally remain after remission. Additionally, the question arises whether vulnerable individuals are less intuitive even before depression breaks out. Therefore, longitudinal designs may be advisable for future research.

### Are there Maladaptive Intuitions in Depression?

In the foregoing, we have considered research and experimental paradigms in which intuition is conceptualized as an adaptive capacity that allows fast coherence detection as well as quick and effortless decision-making (Gigerenzer, 2007; Klein, 2008). For the purpose of a clear demarcation and operationalization of this construct in future research and theorizing in clinical psychology, it would be important to examine intuition and its relation to other depression-related cognitive phenomena. Emotional reasoning, for example, describes the phenomenon to conclude from an emotional reaction that something is proven or true (Beck et al., 1979). It guides decisions and judgments and resembles intuition on a phenomenological level but also regarding the processes it is based on. Both intuition and emotional reasoning have in common that they are influenced by affect, appear automatically and are experienced as self-evident.

The risk to confound intuition with other cognitive phenomena will be illustrated in the following example. Imagine a woman walks down a street and sees two friends sitting in a coffee place. Without thinking about the situation, the woman has the immediate hunch to walk by the café trying to stay unseen. On the one hand one may argue, that this is no example of intuition because the underlying processes were not operating holistically. Using her intuition, the woman would have seen *the bigger picture*. She would have integrated implicit goals and wishes (e.g., the need to interact with other people) into her decision. Moreover, more positive associations regarding those two friends would have been taken into account (Kuhl et al., 2015). This, in turn, may have resulted in the intuitive decision to join the friends. Thus, the reaction of the woman may be interpreted as product resulting from an emotional reasoning process. The current mood state might have influenced the way information was processed (Klein, 1998; Hogarth, 2001; Kahneman and Klein, 2009) and served as evidence for the correctness of the decision (‘it does not *feel* good to join them, therefore I will not join them’; Schwarz, 2002). Thus, from this perspective, the decision rather reflects an automatic decision that followed from emotional reasoning and from the activation of subconscious negative schemes. The access to otherwise adaptive intuitions was, from this point of view, impaired in this example. However, the argument that this was indeed an example of intuition, showing that intuitive decisions and judgments may be biased and flawed is also conceivable. Therefore, future research would do well in disentangling intuition from other emotion- and experience-driven processes influencing decisions and judgments.

Apart from these delimitation problems, it appears to be an important step for future research to examine to what extent intuitions in depressed patients may be influenced by negative distortions and imprints of the implicit memory structure. Along this line, current dual-process models of depression (Beevers, 2005) assume that cognitive vulnerability to depression stems from biased associative, implicit processing (Beevers, 2005; but also see Teachman et al., 2012). Importantly, it is claimed that whenever biased self-referent associative processing remains uncorrected (e.g., when cognitive resources are not available to engage reflective correcting processing) cognitive vulnerability to depression is given (Beevers, 2005). Thus, the question arises whether intuitions may become dysfunctional or unrealistic when they result from biased underlying implicit memory. As biases in implicit memory have mostly been shown in the semantic domain (Watkins, 2002) and especially intuitions based on semantic networks seem to be impaired in depression (Remmers et al., 2015a, 2016a), investigations that connect these two lines of research (e.g., how do implicit memory biases influence intuition?) seem very promising.

## Implications for Clinical Treatment

It is clear from the foregoing that intuitions influence people’s decisions and subsequent actions. On the one hand, the problem in depression may be that patients do not use functional intuitions stemming from holistic information processing (see Kuhl, 2000, 2001). A consequence of this may be that they have difficulties to come to decisions that integrate great amounts of information and reconcile different aspects of the self. Being unable to use these kinds of intuitions may further result in actions and behaviors that are inconsistent with needs, wishes and goals. Moreover, decisions that result from a rather non-integrative process may be experienced as dissatisfying and alienating (see Baumann and Kuhl, 2003). On the other hand, negative self-schemes and dysfunctional core beliefs may not only stabilize depressive symptomatology but may also nourish the development of *dysfunctional* intuitions (Beevers, 2005). Even though, this latter assumption still needs examination, we tentatively conclude that gaining *access* to intuitions may be an important practical implication from the current theorizing. From a practical point of view, establishing awareness of intuitive hunches seems important because this would enable individuals to differentiate between those intuitions that are functional and that may be acted upon and those intuitions that should be dismissed or corrected as they might lead to dysfunctional and depressogenic actions (Shapiro and Spence, 1997; Beevers, 2005). This idea is in line with Kahneman and Klein (2009) positing that ‘when there are cues that an intuitive judgment could be wrong, System 2 [rational-analytic processes] can impose a different strategy, replacing intuition by careful reasoning’ (p. 519). In other words, from a practical point of view, it may be advisable to get aware of and examine intuitions before acting upon or dismissing them.

From a clinical and practical perspective, the question *how* such awareness of intuitions may be enhanced directly follows. Interestingly it strikes out that the wisdom that lies within the every-day expression of ‘go with your gut’ corresponds to a widely established therapeutic conception stating that ‘listening’ to inner voices and to the body may be helpful when we are trying to understand what we need or when we are trying to change what makes us suffer. Along this line, investigations within the scope of embodiment research (Niedenthal, 2007) have shown that the association between the body and cognitive-affective responses is bidirectional. It has further been shown that the degree to which individuals are able to correctly perceive body signals (interoception) influences intuitive decision-making (Damasio, 1994; Dunn et al., 2010). Thus, it may be concluded that it is an important capacity to know which signal (bodily, intuitive) may be trusted and which should be dismissed.

One approach that stresses this aspect of careful listening to bodily experiences is the *Focusing* method introduced by Gendlin (1981). The main premise is that Focusing helps patients to get in touch with the *felt-sense*. The felt sense entails pre-verbal knowledge about ‘something,’ such as what one needs or wants and it may be accessed through the body. The felt sense is not an emotion or a mood state and it entails an implicit complexity. By getting in touch with the felt sense, patients may become more aware of what a difficult situation or a pending decision evokes and they may then gently explore this bodily felt experience and its meaning. In the next step they are encouraged to find a word, phrase, or picture for the bodily felt experience and to examine, whether this word or phrase matches with the not-yet-articulated knowing. If the verbal representation matches with the feeling, the bodily experience generally changes which may be called a *felt shift*. This alteration in the felt-sense may be a result of the preceding process of intuiting and careful examining. As such, the felt sense may be understood as a ‘holistic, implicit, bodily sense of a complex situation’ (Gendlin, 1996, pp. 20) which goes beyond intellectual reflections of a problem.

Another approach by which access to intuitions may be gained via the body is mindfulness. In mindfulness exercises individuals learn to, listen to sensations in the here-and-now in a non-defensive, non-reactive way. Based on the definition of mindfulness as a form of attention that focuses on present feelings, thoughts and bodily sensations (Kabat-Zinn, 1990), we tested the assumption that mindfulness also enhances access to intuitive responses in one of our own investigations (Remmers et al., 2015b). After a sad mood induction, healthy participants (*N* = 94) were randomly assigned to perform either a mindfulness, distraction or rumination exercise. To assess the effect of the respective exercise on intuition, participants then performed the semantic coherence task. Even though mindfulness was successful in down-regulating negative mood, it did not have any impact on the task performance (see Remmers et al., 2015b for a detailed discussion). In addition, it was found that self-reported levels of trait mindfulness, assessed with the Kentucky Inventory of Mindfulness Skills (Baer et al., 2004), were negatively associated with the intuitive performance. As such, the hypothesis was not supported and results even pointed to the opposite direction.

A number of methodological aspects may explain this pattern of findings. For example, the intuition task requires participants to decide and judge instantly. This in turn may stand in contrast to a facet of mindfulness that requires individuals to adopt a non-reactive, non-judgmental, observing attitude. Indeed, results in Remmers et al. (2015b) revealed that the overall negative correlation between trait mindfulness and intuitive accuracy was driven by a strong negative correlation between the *acting without judgment* subscale and the intuitive performance. Furthermore, the sample consisted of subjects who were naïve in mindfulness practice and it has been shown that the degree of mindfulness experience may explain differential effects of trait mindfulness on cognitive tasks (Jha et al., 2007). For example, mindfulness novices train to narrow their attentional focus (attention to the breath) whereas experienced meditators widen their attentional field (Jha et al., 2007). Thus, the low mindfulness experience of the sample may have influenced the pattern of findings in the study of Remmers et al. (2015b).

Another approach that may foster access to intuitive processes is psychodynamic psychotherapy (Shedler, 2010). This approach stems from psychoanalysis of which the central goal was according to Freud (1916/1917) to get access to implicit or unconscious representations and experiences. In line with this, a key focus of psychodynamic treatment is to enhance patients’ access to initially non-conscious knowledge about the self (Hayes et al., 1996). Therefore, it may be concluded that also intuitions are accessed more easily as a consequence of psychodynamic treatment. However, of course the exact relationship between unconscious processes, as defined in psychoanalysis, and intuition as investigated with the experimental paradigms described above has to be determined.

More generally, all treatments mentioned above seem to cultivate a form of self-focus that retains the advantages of self-knowledge (Watkins and Teasdale, 2004, p. 6; see also Kuhl, 2000). As such, it may be assumed that directing attention toward oneself is helpful when being done in a more adaptive manner than during rumination (Watkins and Teasdale, 2004). In such instances, it may enable individuals not to think *about* inner experiences but to get aware of the (self in the) present moment in an intuitive, experiential way (see Watkins and Teasdale, 2004, p. 2). Approaches that foster this kind of experiential self-focus (e.g., the different humanistic-experiential approaches; see Elliott et al., 2013) may create the basic requirements for the access to intuitions. Furthermore, it may be suspected that individuals who have access to intuitions may become aware of subtle conflicts between formerly unconscious, intuitive responses, and conscious elaborations. Resolving such conflicts or discrepancies between intuitive and rational responses may thus be another adaptive consequence of gaining access to intuitions. Indeed, for mindfulness it has been shown in a number of studies that one means by which mindfulness exhibits its beneficial effects is by enhancing the alignment between implicit and explicit responses (Brown and Ryan, 2003; Koole et al., 2009; Crescentini and Capurso, 2015; Remmers et al., 2016b).

As a conclusion, we would like state that intuitions have an impact on what we decide and do and how we subsequently feel. Thus, addressing the question how intuitive decision-making operates during psychopathological states such as depression is an important thrust for science and practice. In the long run, this line of work may help depressed individuals to take adaptive decisions and to find a way out of indecisiveness.

## Author Contributions

CR conceived and wrote the current manuscript. JM made contribution to the design of the paper and revised the manuscript critically for intellectual content. Both authors give their final approvement for the version to be published. All authors take full responsibility for the content of the paper.

## Conflict of Interest Statement

The authors declare that the research was conducted in the absence of any commercial or financial relationships that could be construed as a potential conflict of interest.
